# Diagnosis of behavioral symptoms as a predictor of institutionalization among Medicaid patients with dementia

**DOI:** 10.1186/s12877-023-04506-9

**Published:** 2023-12-05

**Authors:** Rezaul Karim Khandker, Farid Chekani, Kirti Mirchandani, Niranjan Kathe

**Affiliations:** 1 Center of Observational and Real-world Evidence, Merck & Co., Inc, 351 North Sumneytown Pike, North Wales, PA USA; 2 Complete HEOR Solutions (CHEORS), Chalfont, PA USA

**Keywords:** Dementia, Behavioral symptoms, Institutionalization, Cohort study

## Abstract

**Objectives:**

Behavioral symptoms are commonly observed in the course of dementia. This study aimed to assess the association of the diagnosis of a cluster of behavioral symptoms (e.g., agitation, aggression, psychotic symptoms, and delirium/wandering) with the likelihood of subsequent institutionalization.

**Methods:**

A retrospective cohort study of adults aged 65 and above diagnosed with dementia identified in the IBM® MarketScan® Multistate Medicaid database between October 01, 2015, and September 30, 2019, was conducted. The index date was defined as the first diagnosis date of dementia. The presence or absence of behavioral symptoms was identified in the 6 months prior to the index date (baseline). Institutionalization was evaluated 12 months (follow-up) post the index date. The association between diagnosed behavioral symptoms during the baseline period and institutionalization in the follow-up period was assessed using a multivariable logistic regression, adjusting for baseline sociodemographic and clinical characteristics.

**Results:**

The study cohort included 40,714 patients with dementia. A diagnosis of behavioral symptoms was found among 2,067 (5.1%) patients during the baseline period. An increased likelihood of institutionalization was found during the follow-up among patients with agitation and aggression in baseline (OR = 1.51 (95% CI: 1.18–1.92)) compared to patients without these symptoms at baseline. Patients with psychotic symptoms in baseline had significantly higher odds of getting institutionalized during the follow-up compared to patients without psychotic symptoms in baseline (OR = 1.36 (95% CI: 1.20–1.54)). Similarly, patients with symptoms of delirium and wandering in baseline had a higher likelihood of institutionalization than patients without these symptoms at baseline (OR = 1.61 (95% CI: 1.30–1.99)).

**Conclusion:**

Several diagnosed behavioral symptoms were associated with a higher risk of institutionalization among older adults with dementia and should be considered when planning treatment strategies for the effective management of the condition.

**Supplementary Information:**

The online version contains supplementary material available at 10.1186/s12877-023-04506-9.

## Background

Dementia is characterized by a decline in memory and a decrease in at least one area of cognitive function, such as executive abilities, language skills, visuospatial capabilities, praxis, judgment, personality, or abstract thinking that causes interference in occupational, domestic, or social functioning [1]. Aging is a primary risk factor for dementia; therefore, dementia is more prevalent among people aged 65 years and older [2]. Roughly 5 million elderly patients in the United States (US) were suffering from dementia in 2014, which was projected to be tripled by 2060 [3]. Dementia is also one of the leading causes of death among the elderly with approximately 30% of seniors dying from dementia in the US [2]. The dementia-related medical cost was estimated to be approximately 355 billion dollars in 2021 [2]. The total per-person health care and long-term care costs for patients with dementia were also significantly higher than those without dementia [4].

Patients with dementia often display various behavioral or mental symptoms, such as agitation, aggression, psychotic symptoms including hallucinations and delusions, and depression. Delusions and hallucinations in patients with dementia may not necessarily indicate the presence of a psychotic disorder; instead, they are considered psychotic symptoms associated with dementia [1, 5]. A previous study estimated that approximately 56–87% of patients might suffer from one or more behavioral or mental symptoms during the disease [6]. Some of these symptoms, such as agitation and aggression, were often managed through antipsychotics [7, 8]. Until 2023, there was no Food and Drug Administration (FDA) approved medication for managing these behavioral symptoms. However, the FDA has recently approved Rexulti (Brexpiprazole) for the treatment of agitation associated with dementia due to Alzheimer’s disease [9]. The occurrence of behavioral symptoms is associated with rapid cognitive decline [10], and it can further impede daily activities and social functioning [11] and increase the complexity of patient care [12]. These factors may eventually result in a more significant number of physician visits and admission to long-term care facilities [1, 13–15]. Institutionalization in a long-term care facility can impose a substantial economic burden on patients and payers. Total annual cost of institutionalization for individuals with Alzheimer’s dementia was estimated to be over 84 billion dollars in 2018 [16].

Given the economic, health, and social implications of dementia-related behavioral symptoms, it is imperative to understand the extent of diagnosed behavioral symptoms and their potential role in institutionalization. The relationship between behavioral symptoms of dementia and economic outcomes can be influenced by social determinants of health, such as race and type of insurance plan [17]. Current studies that have evaluated these parameters are either based on smaller sample size [18–21] or need to be updated with newer estimates [18, 21–23]. Moreover, there is limited evidence on the risk of institutionalization associated with diagnosed behavioral symptoms sub-types such as agitation/aggression, psychotic symptoms, or delirium/wandering among patients with dementia, especially those covered by Medicaid [24]. Therefore, to fill the evidence gap in the literature, this study aimed to assess the prevalence of diagnosed behavioral symptoms and associated risk of institutionalization among patients with dementia covered by Medicaid. The study hypothesized that these diagnosed behavioral symptoms are associated with a higher risk of institutionalization.

## Methods

### Study design and data source

**Fig. 1:**
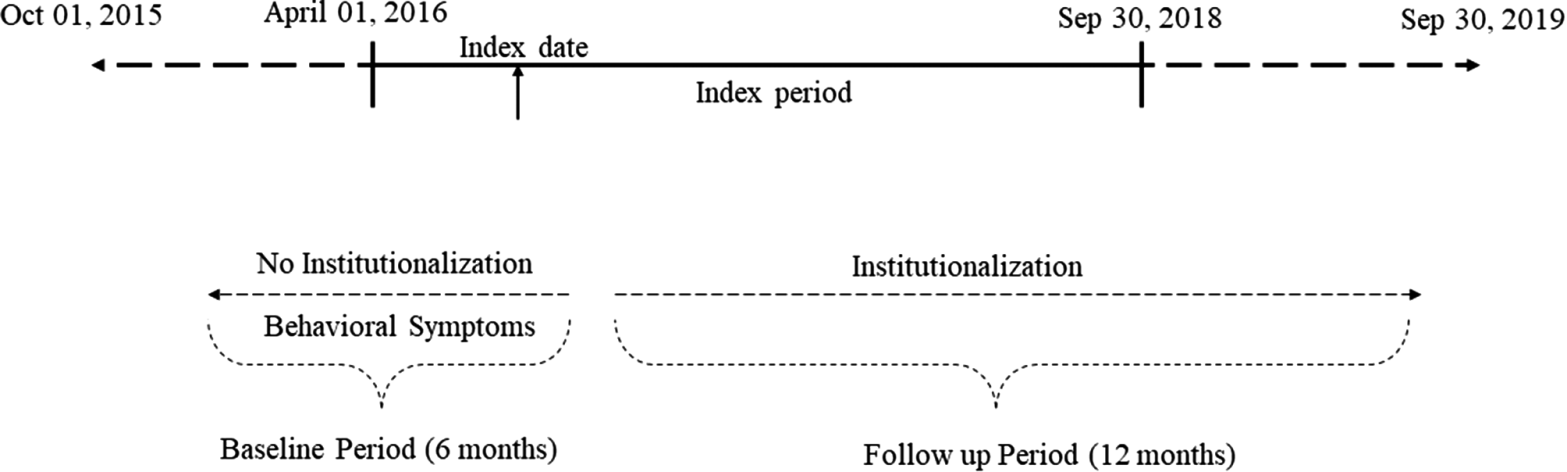
Study design

A retrospective, longitudinal observational study was conducted using the IBM^®^ MarketScan^®^ Multistate Medicaid database [25]. This database, maintained by IBM^®^ Watson Health, belongs to a family of administrative claims databases that have compiled data on approximately 40 million Medicaid enrollees in the United States [26]. The database contains patient-level data on healthcare expenditures, outpatient prescription claims from inpatient and outpatient medical claims, and clinical utilization records. The database also contains demographic details on race and reasons for Medicaid eligibility (disability, financial need, etc.) [26]. The current study used data between October 01, 2015, and September 30, 2019. The patient selection window was April 01, 2016, and September 30, 2018, to allow for a baseline period of 6 months starting October 01, 2015, and a follow-up period of at least 12 months ending on September 30, 2019 (Fig. 1). The study time period was chosen to include the most recent available data and to accommodate the transition from ICD-9 to ICD-10 codes which occurred in October 2015.

### Study measurements

The study cohort consisted of patients with dementia between April 01, 2016, and September 30, 2018, in the IBM Multistate Medicaid database. Patients with dementia were identified as having at least one inpatient or two outpatient diagnoses of dementia during the study period using the International Classification of Disease (ICD), 10th Modification codes [27] (Refer to Supplementary Table 1). The earliest dementia diagnosis date within the patient identification period served as the index date. A prevalent cohort of patients with dementia was utilized as these patients reflect a more generalizable population. The study cohort was restricted to patients aged ≥ 65 years at the index date, having at least 6 months of continuous coverage before the index date, and those who had at least 12 months of continuous coverage after the index date. Continuous coverage was defined as no gap in enrollment greater than 45 days for pharmacy or mental health coverage. Patients with a history of schizophrenia (ICD-10-CM F20.XX, F250, F251, F258, F259) or bipolar disorder (ICD-10-CM F31.XX) in the baseline period were excluded since symptoms may overlap with a diagnosis of behavioral symptoms of dementia.

The presence or absence of behavioral symptoms (agitation/aggression, psychotic symptoms, and delirium/wandering) were assessed during 6 months prior to the index date (baseline period). Further the cohort was divided into two groups: patients with or without these symptoms in the baseline. The list of ICD codes (Refer to Supplementary Table 2) was finalized after a review of clinical research in accordance with previously published studies on dementia focusing on related behavioral disturbance and domains of neuropsychiatric inventory (NPI) [28]. The study population included patients having dementia with or without these behavioral symptoms. The symptoms included in the assessment were those associated with psychotic features of dementia rather than mood disorders. While the cohort excluded bipolar mood disorder, depression was considered a comorbidity and was controlled for as a baseline covariate. To identify individuals with incident institutionalization risk, patients with a history of institutionalization in the baseline period were excluded from the analysis. The presence of institutionalization was further evaluated 12 months after the index date (follow-up period) among patients who satisfied all the inclusion/exclusion criteria. Socio-demographic and clinical characteristics were captured during the baseline period. Social and demographic characteristics included age, sex (Male or Female), index year, plan type (Comprehensive, Health Maintenance Organization (HMO), Preferred Provider Organization (PPO)), and race (White, Black, Hispanic, Missing, and Other). Baseline clinical characteristics included Elixhauser Comorbidity Index (ECI) score, comorbid condition, and selected medications. Comorbidities included hypertension, diabetes, cancer, anemia, congestive heart failure, cardiac arrhythmia, chronic pulmonary disease, renal failure, fluid and electrolyte disorders, and depression. Medications comprised antidepressants, antipsychotics, ASH Benzodiazepines, and Anxiolytic/Sedative/Hypnotic Not Elsewhere Classified (NEC).

### Statistical analyses

Continuous baseline variables were summarized as mean (standard deviation (SD)) and were compared across diagnosed behavioral symptoms groups using the student’s t-test. Categorical variables were summarized as proportions and were compared using the Chi-square test across diagnosed behavioral symptoms groups. Multivariable logistic regression was conducted to assess the association between the diagnosed behavioral symptoms during baseline and institutionalization in the follow-up.

## Results

**Fig. 2:**
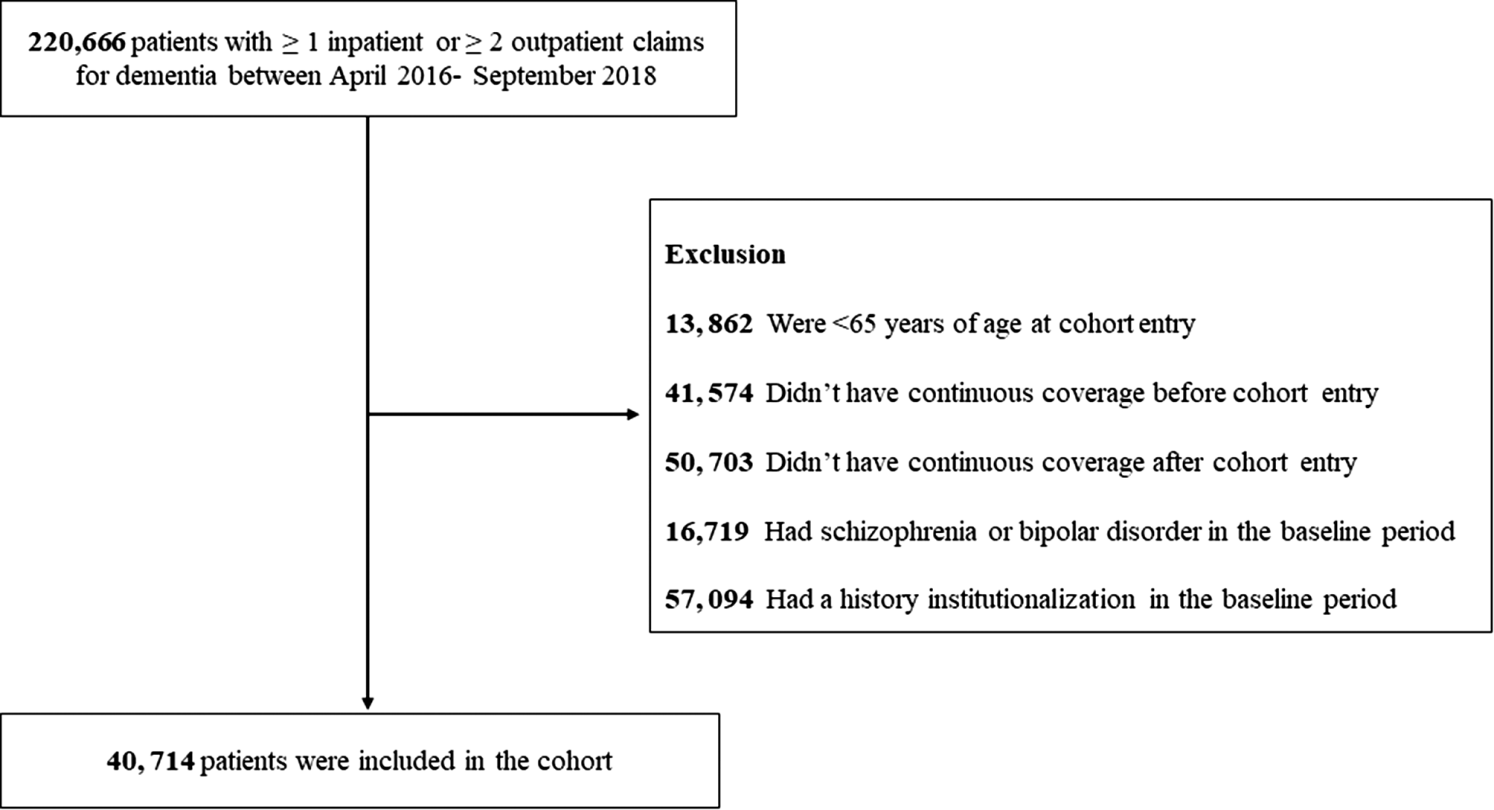
Cohort selection flowchart

A total of 220,666 patients with at least 1 inpatient claim or at least 2 outpatient claims for dementia were identified between April 01, 2016, and September 30, 2018. After applying exclusion criteria sequentially, 179,952 patients were excluded from the analysis, and the final cohort included 40,714 patients with dementia (Fig. 2).

Of 40,714 patients, 2,067 (5.1%) had diagnosed behavioral symptoms during the baseline period. Table 1 summarizes the baseline patient characteristics according to the occurrence of diagnosed behavioral symptoms during the baseline. Table 1 shows that age, race and several comorbidities were significantly different between the cohort of patients with behavioral symptoms at baseline versus those without. These results indicated the need for multivariate modeling of these predictors in explaining institutionalization during the follow-up period.

Among patients with diagnosed behavioral symptoms during the baseline period, 1,368 (66.2%) patients were diagnosed with psychotic symptoms, 403 (19.5%) patients were diagnosed with delirium and wandering, and 325 (15.7%) patients were diagnosed with agitation/aggression. Overall, 9,137 (22.4%) patients were institutionalized during the follow-up among 40,714 patients without a history of institutionalization. Among patients with diagnosed behavioral symptoms in the baseline period, 638 (30.9%) were institutionalized during the follow-up period. Among patients without these symptoms at baseline, 8,499 (22%) patients were institutionalized during the follow-up period (Table 2).

**Table 1:** Baseline patient characteristics of patients with/without diagnosed behavioral symptoms in the baseline period

Characteristics N (%)	With behavioral symptoms in baseline (N = 2,067)	Without behavioral symptoms in baseline (N = 38,647)	p-value*
Age (Mean (SD))	79.7 (8.5)	81.2 (7.9)	< 0.0001
Age group, N (%)			
65–74	639 (30.9%)	9,081(23.5%)	
75–79	377 (18.2%)	6,546 (16.9%)	<0.0001
80–84	345 (16.7%)	7,878 (20.4%)	
85+	706 (34.2%)	15,142(39.2%)	
Sex, N (%)			
Male	528 (25.5%)	9,346 (24.2%)	
Female	1,539 (74.5%)	29,301 (75.8%)	0.160
Plan type, N (%)			
Comprehensive	1,749 (84.6%)	33,286 (86.1%)	
HMO	309 (14.9%)	5,253 (13.6%)	
PPO	9 (0.4%)	108 (0.3%)	0.090
Race, N (%)			
White	1,216 (58.8%)	20,178 (52.2%)	
Black	659 (31.9%)	13,576 (35.1%)	
Hispanic	38 (1.8%)	930 (2.4%)	
Missing	32 (1.5%)	697 (1.8%)	< 0.0001
Other	122 (5.9%)	3,266 (8.5%)	
ECI (Mean (SD))	5.9 (4.71)	4.1(4.41)	< 0.0001
Comorbidities, N (%)			
Congestive heart failure	423 (20.5%)	5,500 (14.2%)	< 0.0001
Cardiac arrhythmias	494 (23.9%)	6,215 (16.1%)	< 0.0001
Valvular disease	173 (8.4%)	2,278 (5.9%)	< 0.0001
Pulmonary circulation disorders	72 (3.5%)	809 (2.1%)	< 0.0001
Peripheral vascular disorders	336 (16.3%)	4,795 (12.4%)	< 0.0001
Hypertension, uncomplicated	1,488 (72.0%)	24,086 (62.3%)	< 0.0001
Hypertension, complicated	305 (14.8%)	4,285 (11.1%)	< 0.0001
Paralysis	81 (3.9%)	646 (1.7%)	< 0.0001
Other neurological disorders	633 (30.6%)	5,438 (14.1%)	< 0.0001
Chronic pulmonary disease	536 (25.9%)	7,810 (20.2%)	< 0.0001
Diabetes, uncomplicated	665 (32.2%)	11,193 (29.0%)	0.002
Diabetes, complicated	453 (21.9%)	7,356 (19.0%)	0.001
Hypothyroidism	371 (17.9%)	4,832 (12.5%)	< 0.0001
Renal failure	336 (16.3%)	5,153(13.3%)	0.000
Liver disease	62 (3.0%)	692 (1.8%)	< 0.0001
Peptic ulcer disease excluding bleeding	23 (1.1%)	192 (0.5%)	0.000
AIDS/HIV	2 (0.1%)	72 (0.2%)	0.352
Lymphoma	7 (0.3%)	189 (0.5%)	0.336
Metastatic cancer	5 (0.2%)	227 (0.6%)	0.042
Solid tumor without metastasis	87 (4.2%)	1,792 (4.6%)	0.366
Rheumatoid arthritis/collagen vascular diseases	67 (3.2%)	1,162 (3.0%)	0.543
Coagulopathy	68 (3.3%)	771 (2.0%)	< 0.0001
Obesity	133 (6.4%)	1,606 (4.2%)	< 0.0001
Weight loss	209 (10.1%)	2,236 (5.8%)	< 0.0001
Fluid and electrolyte disorders	527 (25.5%)	4,784 (12.4%)	< 0.0001
Blood loss anemia	34 (1.6%)	396 (1.0%)	0.007
Deficiency anemia	204 (9.9%)	2,595 (6.7%)	< 0.0001
Alcohol abuse	81 (3.9%)	539 (1.4%)	< 0.0001
Drug abuse	61 (3.0%)	371 (1.0%)	< 0.0001
Psychoses	693 (33.5%)	189 (0.5%)	< 0.0001
Depression	736 (35.6%)	5,615 (14.5%)	< 0.0001
Medications, N (%)			
Antidepressants use	262 (12.7%)	3,201 (8.3%)	< 0.0001
Antipsychotics use	182 (8.8%)	891(2.3%)	< 0.0001
ASH, Benzodiazepines use	97 (4.7%)	945 (2.4%)	< 0.0001
Anxiolytic/Sedative/Hypnotic NEC	47 (2.3%)	508 (1.3%)	0.000

AIDS: Acquired Immunodeficiency Syndrome; ASH: Anxiolytic/Sedative/Hypnotic; ECI: Elixhauser Comorbidity Index, HMO, Health Maintenance Organization; HIV, Human Immunodeficiency Virus; NEC: Not Elsewhere Classified; PPO, Preferred Provider Organizations; SD: Standard Deviation

*χ2 Tests (or Fisher’s exact test) for categorical variables, and t tests for continuous variables were used to calculate p-value which evaluates the differences in patient characteristics between patients with and without behavioral symptoms in baseline

Table 3 presents the results from the multivariate logistic regression predicting institutionalization in the follow-up period. Various covariates (socio-demographic and clinical comorbidity variables) as well as the presence or absence of specific behavioral symptoms sub-types identified during the baseline period were used as predictors. As hypothesized, individual behavioral symptoms categories were significant predictors of institutionalization. Specifically, while controlling for other covariates, an increased likelihood of institutionalization was found among the patients with agitation and aggression at baseline [Odds Ratio (OR): 1.51; 95% Confidence Interval (CI): 1.18–1.92] compared to patients without agitation/aggression at baseline. Patients with psychotic symptoms in the baseline period had significantly higher odds of getting institutionalized in the follow-up than patients without psychotic symptoms at baseline (OR: 1.36; 95% CI: 1.20–1.54). Similarly, patients with symptoms of delirium and wandering had a significantly higher likelihood of getting institutionalized than patients without delirium and wandering at baseline (OR: 1.61; 95% CI: 1.30–1.99). Moreover, increased age, being white, male, and being enrolled in an HMO were associated with increased risk of institutionalization. In addition, patients having comorbid conditions such as diabetes, anemia, renal failure, and congestive heart failure were less likely to be institutionalized. Conversely, patients having hypertension, cancer, chronic pulmonary disease, cardiac arrhythmia, fluid and electrolyte disorders, depression, and higher ECI were more likely to be institutionalized (Table 3). Although not shown here, all of the behavioral symptoms were combined and run in a separate multivariable logistic regression. The analysis revealed that patients with diagnosed behavioral symptoms in baseline were 1.45 (OR: 1.45; 95% CI: 1.31–1.60) times as likely to get institutionalized in the follow-up period compared to patients without these behavioral symptoms in baseline. This finding led to the choice of running the final regression reported here where separate binary predictor variables were used for each of the three behavioral symptom subtypes.

**Table 2:** Proportion of institutionalization in the follow-up period among patients with and without diagnosed behavioral symptoms

N (%)		Institutionalization in follow-up	
		Yes	No
Behavioral symptoms in baseline	Yes	638 (30.9)	1,429 (69.1)
	No	8,499 (22)	30,148 (78)
Total		9,137	31,577

**Table 3:** Logistic regression results of the likelihood of institutionalization during the follow-up among patients with no history of institutionalization

Characteristics	Odds Ratio	95% Confidence Interval	p-value
**Age Group (Years)**			
65–69	1.00	Reference	Reference
70–74	1.13	1.01–1.25	0.0218
75–79	1.20	1.09–1.32	0.0004
80–84	1.30	1.18–1.43	< 0.0001
85+	1.48	1.35–1.62	< 0.0001
**Year**			
2016	1.00	Reference	Reference
2017	1.30	1.23–1.37	< 0.0001
2018	1.36	1.19–1.56	< 0.0001
**Race**			
White	1	Reference	Reference
Black	0.65	0.62–0.69	< 0.0001
Hispanic	0.37	0.31–0.45	< 0.0001
Other	0.26	0.23–0.29	< 0.0001
Missing	0.31	0.25–0.39	< 0.0001
**Sex**			
Female	1.00	Reference	Reference
Male	1.17	1.11–1.24	< 0.0001
**Plan type**			
Comprehensive	1.00	Reference	Reference
HMO	1.78	1.67–1.91	< 0.0001
Other	0.85	0.41–1.76	0.660
**Comorbidities**			
Hypertension	0.86	0.81–0.91	< 0.0001
Diabetes	0.99	0.94–1.04	0.637
Cancer	0.83	0.74–0.93	0.002
Anemia	0.93	0.85–1.02	0.118
Congestive Heart Failure	1.02	0.94–1.10	0.696
Cardiac Arrhythmia	1.09	1.01–1.17	0.018
Chronic Pulmonary Disease	0.93	0.88-1.00	0.035
Renal Failure	1.08	1.00-1.16	0.062
Fluid and Electrolyte Disorders	1.19	1.10–1.28	< 0.0001
Depression	1.08	1.01–1.15	0.027
ECI	1.03	1.02–1.04	< 0.0001
**Medications**			
Antidepressant Medications	1.00	0.91–1.10	0.976
Antipsychotic Medications	1.17	1.01–1.36	0.036
ASH Benzodiazepines	0.91	0.78–1.06	0.217
Anxiolytic/Sedative/Hypnotic NEC	1.11	0.91–1.35	0.310
**Baseline behavioral symptom sub-types**			
Agitation and Aggression in baseline	1.51	1.18–1.92	0.001
Psychotic symptoms in baseline	1.36	1.20–1.54	< 0.0001
Delirium and wandering in baseline	1.61	1.30–1.99	< 0.0001

ASH: Anxiolytic/Sedative/Hypnotic; ECI: Elixhauser Comorbidity Index, HMO, Health Maintenance Organization; NEC: Not Elsewhere Classified; PPO

## Discussion

In this study, we estimated the association of diagnosis of a set of behavioral symptoms (agitation and aggression, psychotic symptoms, delirium, and wandering) found in claims-coded data and their association with institutionalization among Medicaid-insured US population aged ≥ 65 years and diagnosed with dementia. In claims data, the frequency of these symptoms was rather low (only around 5%). The prevalence of these symptoms was reported to be 90% throughout the illness [24, 29]. The estimates vary from 35 to 85% in patients with mild cognitive impairment, which is quite a wide range [30]. The recent findings from Chekani et al. estimated the prevalence of behavioral symptoms to be approximately 81% among dementia patients using data from 2015/16 Adelphi Real World Dementia Disease-Specific Programme™ [31]. This difference in numbers can be attributed to the fact that our study identified diagnosed behavioral symptoms subtypes, namely agitation/aggression, psychotic symptoms, and delirium/wandering, for only a short duration of 6 months and used claims data compared to published literature where studies have used data sources other than claims and different definitions of behavioral and neurological disorders, for e.g., using the neuropsychiatric inventory (NPI) scale [5]. The NPI of Cummings (1994) is an informant-based survey instrument with 12 subscales: delusions, hallucinations, agitation, depression, anxiety, elation, apathy, disinhibition, irritability, aberrant motor behavior, sleep disorders, and appetite [32]. The relationship between the NPI and diagnosed behavioral symptoms is not well-studied. NPI scale may be more sensitive in capturing mild behavioral symptoms compared to claims-based algorithm. In contrast, claims-based diagnosis codes only identify those patients with relatively severe symptoms that require medical assistance. Thus, less severe cases of behavioral symptoms may not have been captured. Furthermore, the low rates may also be due to the under-coding of the behavioral symptoms in claims data.

Cejeria et al. highlighted the lack of uniformity in assigning clusters of behavioral symptoms in prior studies [24]. An example of a cluster studied in the literature includes behavioral dyscontrol (i.e., euphoria, disinhibition, aberrant motor behavior, sleep, appetite), psychosis (i.e., delusions, hallucinations), mood (i.e., depression, anxiety, apathy), agitation (i.e., irritability, aggression) [33]. Another example of a cluster studied in recent literature includes mood (i.e., apathy, depression/euphoria), psychosis (i.e., delusions, hallucinations, anxiety, agitation, disinhibition, irritability, aberrant motor activity), and euphoria [34]. Aigbogun et al. considered a single ICD-9 code of agitation as a proxy to describe behavioral symptoms in general [35]. While our study may not capture all possible categories of behavioral symptoms, it fills an important gap in the literature by assessing the association of certain diagnosed behavioral symptoms with institutionalization. Several studies have emphasized the importance of selected behavioral symptoms, especially, aggression, agitation, hallucination, and delusion, as independent risk factors leading to institutionalization [13, 36]. In this study, the selected behavioral symptoms are those which are observed as psychotic features such as delusions, hallucinations, irritability, and aggression [37]. These behavioral symptoms are the leading cause of institutionalization for patients with dementia [13, 36]. Assessing factors associated with the risk of institutionalization to a long-term care facility may help plan appropriate screening and management strategies to prevent future institutionalization among patients with dementia.

About 22% of the entire study sample was institutionalized during the study follow-up, and the proportion was much higher among those who had diagnosed behavioral symptoms at baseline compared to those without diagnosed behavioral symptoms. After adjusting for baseline covariates, the behavioral symptoms subtypes studied here were all significantly associated with institutionalization. In addition to diagnosed behavioral symptoms subtypes, several other baseline characteristics were also associated with institutionalization. Older age was associated with a higher risk of institutionalization. This is in line with prior knowledge, including a recent European study that reported older individuals were more likely to be institutionalized than younger ones over the course of a three-year follow-up [38]. Black or Hispanic race was associated with a lower likelihood of institutionalization and aligned with a previous report [23]. The lower likelihood might be attributed to the disparities experienced by these patients in terms of availability of long-term care beds, insufficient family support, etc.

To our knowledge, this study is the first to estimate the association of diagnosed behavioral symptoms with institutionalization among a national sample of Medicaid-insured patients diagnosed with dementia in the US. This dataset was chosen for the study as the information regarding the institutionalization to a long-term care facility is included in the Medicaid dataset. Previous research conducted using the National Alzheimer’s Coordinating Center Uniform Data Set (NACC-UDS) cohort demonstrated that patients with agitation were 20% more likely to be institutionalized compared to patients without agitation (OR: 1.20; 95% CI: 1.08–1.33) [16]. The differences in the strength of association between the previous study and the current study are likely due to differences in age and racial composition of the two study cohorts and the methods used to identify patients with agitation. Another study by Park et al. used clinical measures and medication categories but did not separate individual categories of behavioral symptoms. The study findings highlighted that patients with Alzheimer’s disease having lower cognitive ability, higher dementia severity, and more-severe behavioral symptoms at baseline were more likely to be institutionalized earlier which aligns with the findings of our study [39]. Another study found that caregiver distress related to patients with behavioral symptoms was a significant predictor of nursing home placement, while behavioral symptoms were not [18]. While the caregiver distress related to the patient behavioral symptoms could be an important factor in the decision to institutionalize the patient [18], it is also likely a mediator between behavioral symptoms and institutionalization. Therefore, a mediator in the model may have attenuated the association between behavioral symptoms and institutionalization [40].

A relatively common occurrence of behavioral symptoms among older adults with dementia, its strong association with the risk of institutionalization [15, 19, 20], and the high annual incremental cost of institutionalization [16] are likely the driving factors for high healthcare expenditures among this patient population. Indeed, according to 2021 estimates, the direct economic burden of dementia from the Center for Medicare and Medicaid Services (CMS) is estimated to be 239 billion USD [2], and this cost may further increase with the aging population [41]. In addition, the occurrence of behavioral symptoms may have other social and clinical consequences, such as caregiver stress due to the disruptive and unsafe nature of behavioral symptoms [42, 43] and the increasing complexity of managing such patients [44]. These may, in turn, contribute to further deterioration of dementia and behavioral symptoms. Despite these substantial financial, social, and clinical consequences of behavioral symptoms among patients with dementia, pharmacological treatments for behavioral symptoms are yet to be approved in the US. The current standard of care for patients with dementia with diagnosed behavioral symptoms consists of non-pharmacological treatments such as behavioral, environmental, and caregiver supportive interventions and off-label use of specific pharmacological products such as antipsychotics, anticonvulsants, and antidepressants [45]. Considering the current unmet need of patients with dementia with behavioral symptoms, pharmacological interventions may be needed to alleviate behavioral symptoms among patients with dementia.

This study used a national sample of Medicaid enrollees from geographically diverse State Medicaid programs in the United States. This study has some limitations. First, given this study relied on claims data involving prevalent patients; it was difficult to determine how long patients had been living with dementia. Some patients with behavioral symptoms might have had undiagnosed dementia before their symptoms were recognized or reported. Moreover, due to the observational nature of this study, causation cannot be implied between presence of behavioral symptoms in baseline and subsequent institutionalization in the follow-up. While we included comorbidities as covariates for risk adjustment and identified a statistically significant association between behavioral symptoms in baseline and institutionalization in follow-up, we must acknowledge that other unmeasured factors or confounding variables may still be influencing this relationship. Therefore, caution should be exercised when interpreting these findings as causation cannot be inferred. Residual confounding likely persists, either due to incorrect functional form of measured confounders or due to unmeasured confounders. History of dementia is likely associated with the development of behavioral symptoms and the risk of institutionalization, which may confound the estimated association. Furthermore, behavioral symptoms (exposure) and institutionalization (outcome) were identified using diagnosis and procedural codes, which may be prone to misclassification. However, such potential misclassification would likely move the association towards the null [46].

## Conclusion

Behavioral symptoms diagnosed among older patients with dementia (aged ≥ 65 years) in the US Medicaid population were associated with a higher risk of institutionalization. Concerted efforts are needed to manage behavioral symptoms among patients with dementia to reduce the clinical, humanistic, and economic burden. With currently limited treatment options for patients with dementia and behavioral symptoms, research and development efforts may be needed to alleviate behavioral symptoms and associated healthcare burden among patients with dementia.

### Electronic supplementary material

Below is the link to the electronic supplementary material.

Supplementary Material 1

Supplementary Material 2

## Data Availability

The data supporting this study’s findings are available from the IBM® MarketScan® Commercial Claims and Encounters database. However, restrictions apply concerning their availability, which was used under license for the current research and is not publicly available. However, data are available from the corresponding author upon reasonable request and with permission from IBM® Watson Health™.

## List of abbreviations

CI: Confidence Interval
ECI: Elixhauser Comorbidity Index
ICD: International Classification of Disease
NPI: Neuropsychiatric Inventory
OR: Odds Ratio
SD: Standard Deviation
